# Can Nanoplastics Alter Cell Membranes?

**DOI:** 10.1002/cphc.201900481

**Published:** 2019-09-16

**Authors:** Oldamur Hollóczki, Sascha Gehrke

**Affiliations:** ^1^ Mulliken Center for Theoretical Chemistry University of Bonn Beringstr. 4+6 53115 Bonn Germany

**Keywords:** environmental effects, membranes, molecular dynamics, nanoplastics, phospholipid bilayers

## Abstract

Whilst the formation of plastic nanoparticles (nanoplastics) from plastic wastes has been unequivocally evidenced, little is known about the effects of these materials on living organisms at the subcellular or molecular levels. In the present contribution we show through molecular dynamics simulations that polyethylene nanoparticles dissolve in the hydrophobic core of lipid bilayers into a network of disentangled, single polymeric chains. The thereby induced structural and dynamic changes in the bilayer alter vital functions of the cell membrane, which if lacking a mechanism to decompose the polymer chains may result in the death of the cell.

## Graphical abstract

**Fantastic plastics**? Nanoplastics were found to dissolve in lipid bilayers through disentangling into uncoiled polymer chains. The changes in the bilayer structure and dynamics suggest that nanoplastics can have severe effects on cell membranes and thereby on the environment as well.

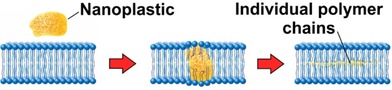

The abundance of plastic waste in the form of sub‐millimeter sized particles (microplastics) in the environment raised significant scientific and public concern in the last decades.^1^, ^2^, ^3^ The extent of the hazard microplastics pose to living organisms is currently under debate,^4^ which clearly shows the underlying challenges in the corresponding research. There seems to be, however, a general consensus that the even smaller (<100 nm) particles, nanoplastics, potentially pose a greater threat to the environment both in terms of exposure and effects.^4^, ^5^, ^6^ Such nanoparticles are at least two orders of magnitude smaller than eukaryote cells, and they are likely to influence biological systems at the subcellular, or even at the molecular level. Nonetheless, the technical difficulties in investigating particles of this size in complex biological media have hindered obtaining sufficient amount of reliable data on this matter, and the related information in literature is scarce.^4^, ^5^, ^6^, ^7^ It has been suggested that the investigation of this issue could be facilitated tremendously, if it would rely more on a material sciences approach,^4^, ^7^ which has been proven to be useful in uncovering the physicochemical behavior of other nanometer‐sized materials, e. g. metal nanoparticles. In these efforts, theoretical and simulation methods, especially molecular dynamics simulations, have been of extraordinary use,^8^, ^9^, ^10^, ^11^ hence they may also aid revealing the molecular level interactions between nanoplastics and well‐defined, biologically relevant systems. Nonetheless, understanding the physicochemical processes that govern the interactions between nanoplastics and living matter has not been attempted by using these modeling tools.

As the first proof‐of‐concept study of this endeavor, we aim here to uncover effects of nanoplastic particles (or plastic nanoparticles, PNPs) on the cell membrane through molecular dynamics simulations. The cell membrane has multiple functions in the cell, including its roles in signaling, energy production processes, while separating the cell from its environment. Thus, if the subcellular and molecular level effects of PNPs on living organisms are to be understood, effects on the cell membrane are essential to reveal. The structure of cell membranes is governed mainly by the self‐organization of hydrophobic and hydrophilic moieties of their components, forming a hydrophilic outer surface and a hydrophobic core. Accordingly, the hydrophobic nature of plastics will expectedly play an important role in these interactions. Fullerenes (C_60_) were shown to dissolve spontaneously in the hydrophobic core of cell membranes, and through changing the properties of the membrane this process resulted in the death of the cell.^10^, ^11^ Furthermore, in an extensive simulation study the diffusion of fullerenes C_60_–C_540_ (with diameters up to 2.5 nm) into the hydrophobic core of phospholipid bilayers was shown to be thermodynamically favorable, and to occur without any barrier despite the presence of the charged head groups of the lipid molecules.11b In terms of hydrophobicity fullerenes and PNPs are similar, and therefore the dissolution of the latter compounds can also be expected in the hydrophobic core of the lipid bilayer. However, since they are composed of loosely entangled polymer chains that are held together by weak non‐covalent interactions, PNPs may alter their size and shape. This expectedly characteristic property of PNPs may enable a richer portfolio of interactions and thereby a plethora of biological effects, while it should also facilitate their diffusion into or through cell membranes. In agreement, PNPs of various sizes were shown to absorb in scallops, reaching all organs of the animal, which directly proves the capacity of these particles to diffuse through cell membranes.^6^

Thus, according to the reasoning above, it is reasonable to assume a spontaneous diffusion of a PNP into the hydrophobic core of the lipid bilayer. Hereby we simulated a globular polyethylene nanoparticle with a 5 nm diameter, situated within a POPC bilayer as a transmembrane object. In this simulation, the lipid molecules spontaneously rearrange to cover more of the plastic, reducing the interface between the hydrophobic polyethylene and water, while the interface between the hydrophobic plastic and the hydrophobic moieties of the lipids can be increased. The change in the shape of the nanoparticle is conspicuous, when comparing the calculated total surface of the PNP (ca. 11000 Å^2^, Figure 1) to that in reference calculations of the same particles in an aqueous solution in the absence of the lipids (6307 Å^2^). The data reveals that due to the interactions with the membrane the surface of the PNP almost doubles. This massive change in the shape of the PNP shows that the present particle adjusts to its environment, the membrane.

**Figure 1:**
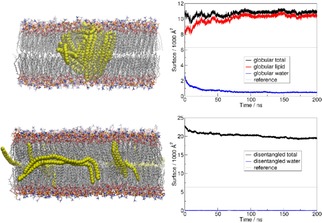
Snapshots of the polyethylene nanoparticle as a globular (top), and a disentangled object (bottom) within the POPC bilayer in our simulations (polyethylene: yellow; lipid C: grey; O: red; P: yellow; N: blue; water molecules and NaCl are omitted for clarity). The time development of the surface of the plastic particle, and that of its contact interfaces with other species is shown on the right for the globular particle. In case of the disentangled chains the interface with water remains very close to 0 throughout the simulation, making the total surface of the chains and their interface with the lipids very similar. The references are given for the water‐PNP interface in an aqueous solution.

The results described above can be rationalized through the cell membrane aiding the disassembly of the PNP into smaller structures, even into individual chains. Having observed this effect, we also simulated a system, in which linear chains of polyethylene were distributed in between two POPC leaflets. The chains stay mostly disentangled, their slight aggregation occurs without any coiling, forming a bundle of parallel oriented polymers within the hydrophobic core of the membrane (Figures 1 and 2). While a large portion of these molecules are oriented parallel also to the membrane surface, their terminal sections apparently move somewhat more freely, pointing out of this almost two‐dimensional space.

**Figure 2:**
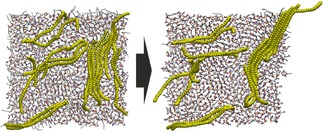
Snapshots of the simulation box of disentangled polyethylene chains in a POPC bilayer after equilibration (left) and after 200 ns of production run (right). For color code, see Figure 1.

According to the data above, in smaller concentrations, polyethylene PNPs are expected to disintegrate, and thereby removed from the environment. This is particularly interesting in the light of recent reports, describing bacteria that can metabolize polyethylene in the digestive tract of a species of moths.^12^ Despite the direct technological impact of these discoveries, the exact mechanism of the metabolism is far from being understood. One of the open questions is how the plastic, chewed by the moth into small particles, can be absorbed by the individual cells of bacteria, and how the enzymes can decompose the polymer pieces. The disintegration of the plastic pieces into linear structures by the membrane might be the key that enables the further steps of the metabolism by making the plastic more digestible for the related enzymes.

Compared to a neat POPC phospholipid bilayer in the absence of the plastic, the change in the overall structure of the membrane can be observed. The conformation of lipid molecules is affected by the PNP. Compared to the neat bilayer, the radial distribution functions of intramolecular distances between the phosphorus atom and the terminal carbon atoms of the lipid side chains show a significant enrichment in the longer distances upon the addition of the plastic in either forms (Figure 3A and 3B). On the other hand, significant depletion of the shorter P−C distances can be observed, except for a single peak in the unsaturated oleoyl at 5 Å in the presence of the globular PNP. These changes are consistent with the stretching of lipid hydrocarbon chains to more linear conformations. As a result of these altered conformations, upon the incorporation of the plastic into the membrane, the thickness of the bilayer−measured as the average distance between the phosphorus atoms in the hydrophilic head groups in the two layers of lipids−increases significantly, by approximately 1.1 nm (ca. 27 %, Figure 3C). As the two non‐polar side chains of the lipid become more linear, lipids can approach each other closer, and the average area per lipid decreases dramatically (Figure 3D).

**Figure 3:**
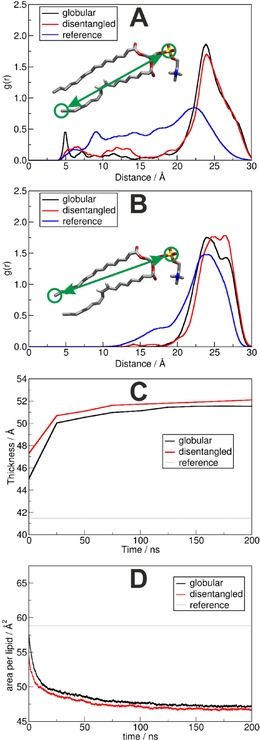
Conformational changes in the POPC phospholipid oleoyl (A) and palmitoyl (B) chains in the lipid bilayer, induced by the presence of the globular polyethylene plastic nanoparticle (black) or disentangled polymer chains (red) in the last 10 ns of the simulations. C) The conformational changes gradually increase the thickness of the membrane (defined by the distance of the phosphorus atoms in the two layers) and D) the average area per lipid decreases over the course of the 200 ns molecular dynamics simulation.

Beyond the structural properties of the cell membrane, dynamic features also play an important role in fulfilling its functions. Recently it has been shown that anesthetics act through changing these very characteristics.^13^ It is therefore vital to see if the presence of the nanoplastic particle alters the dynamics within the lipid bilayer. The dynamics of the membrane were investigated here first through the mean square displacement of the center‐of‐mass of the lipids. Surprisingly, the resulting diffusion constants show a distinct increase in the presence of the plastics (globular: *D*=0.52 pm^2^ ps^−1^; disentangled: *D*=0.72 pm^2^ ps^−1^) compared to the neat bilayer (*D*=0.37 pm^2^ ps^−1^). Thus, the PNP facilitates the movement of lipids within the bilayer.

Thus, all observables suggest that PNPs have a significant effect on the membrane. The changes in its thickness and in its dynamics indicate that the diffusion of molecules through the surface might be significantly altered by the presence of PNPs. Thus, while in small concentrations the disintegration of the PNP into individual polymer chains can be expected in a manner that the plastic may be completely decomposed, at higher concentrations the alterations in the membrane functions may be so severe that cell death is induced, unless the cell has the ability to decompose these chains, and thereby keep their concentration low.

Thus, potential environmental effects of plastic wastes reach the subcellular level, which will be investigated in future forthcoming studies. Consequently, the present results indicate that there is an urgent need to further explore the interactions of nanoplastics and biomolecular structures, in which modeling can be of great help.

## Experimental Section

The periodic simulation box with the globular PNP were created with a single nanoparticle as a transmembrane object in a lipid bilayer that contained 2×300 molecules of 1‐palmitoyl‐2‐oleoylphosphatidylcholine (POPC). 25000 water molecules, 70 Na and 70 Cl ions were added to the system that constitutes a 0.145 M physiological saline solution. The spatial arrangement of the molecules within the orthorhombic periodic simulation box was set up in a manner that the lipids formed an infinite, continuous bilayer within the aqueous solution. In a separate box, a double layer of 2×324 lipids were build, with 16 C_72_H_144_ polymer chains oriented randomly in between the two layers. The system was completed by the addition of 25000 water molecules, 70 Na and 70 Cl ions. In addition to these, further simulation boxes were created for simulating the lipid bilayer and the saline solution in the absence of the nanoparticle, and the nanoparticle in the saline solution in the absence of the lipid bilayer as well.

United atom force field parameters of lipids and plastics were obtained from the Automated Topology Builder and Repository (ATB) version 3.0,^14^ for water the SPC/E model was employed.^15^ These models have all been validated and used repeatedly in the modeling of biomolecules in general,^14^ and cell membranes in particular.^16^ Molecular dynamics simulations and energy minimizations were performed by using the LAMMPS program package.^17^ The 5 ns equilibration and the subsequent 200 ns production runs were performed at 293 K temperature and 1 bar pressure. The analysis of the trajectories was performed by the TRAVIS program.^18^ The surface coverages were calculated by using the Voronoi tesselation‐based domain analysis function of TRAVIS.^19^ For further details on the simulations, see the Supporting Information.

## Conflict of interest

The authors declare no conflict of interest.

## Supporting information

As a service to our authors and readers, this journal provides supporting information supplied by the authors. Such materials are peer reviewed and may be re‐organized for online delivery, but are not copy‐edited or typeset. Technical support issues arising from supporting information (other than missing files) should be addressed to the authors.

Supplementary
