# Supplementary material for: Can Nanoplastics Alter Cell Membranes?
Source: Chemphyschem. 2019 Sep 16;21(1):9–12. doi: 10.1002/cphc.201900481 (PMC6973106; doi:10.1002/cphc.201900481)
Supplement: Supplementary file 1 — Supplementary [file CPHC-21-9-s001.pdf]

CHEMPHYSCHEM

Supporting Information

© Copyright Wiley-VCH Verlag GmbH & Co. KGaA, 69451 Weinheim, 2019

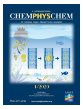

Can Nanoplastics Alter Cell Membranes?

Oldamur Hollóczki* and Sascha Gehrke© 2019 The Authors. Published by Wiley-VCH Verlag GmbH & Co. KGaA. This is an open access article under the terms of the Creative Commons Attribution License, which permits use, distribution and reproduction in any medium, provided the original work is properly cited.

Supporting Information

The plastic nanoparticles were created from single polymer chains of the corresponding plastics through a series of molecular dynamics simulation and energy minimization steps. For polyethylene 16 pieces of $C_{72}(H_{146})$ chains were taken. The hydrogen atoms in the system are implicitly included through the force field that was employed.

The chains with an initial fully linear conformation were simulated in an NpT ensemble employing Nosé–Hoover chain thermostat and barostat at 500 K and under 1 bar pressure for 5 ns, in order to generate a random alignment of the polymer chains with respect to each other. From the 5 ns trajectory 10 structures were collected with 0.5 ns increments for each plastics. These structures were placed into a larger cubic box with cell vectors of 100 Å, containing only the plastic chains. These simulation boxes were simulated in an NVT ensemble by using Nosé–Hoover chain thermostat at three different temperatures (300 K, 400 K, and 500 K) each for 1.5 ns, after which they were cooled down gradually to 200 K over a 10 ns molecular dynamics run. An energy minimization was performed on each of the resulting 15 structures, and the total energies were compared. For the subsequent molecular dynamics simulations the most stable structure (i.e. that with the lowest total energy) was chosen.

All molecular dynamics simulations and energy minimizations were performed by using the LAMMPS program package.[16] The simulation boxes that were created with the lipid bilayers were simulated in an NpT ensemble at 293 K and under 1 bar pressure by using a Nosé–Hoover chain thermostat and barostat. To match the anisotropy of the system, anisotropic barostat was used. After 1 ns of equilibration, the system was simulated for 50 ns, with saving the atomic positions in every picosecond.

The systems with the plastic nanoparticles in the aqueous solutions were simulated for 1 ns in an NpT ensemble with the same settings as above, but using an isotropic barostat. The volume of the cell was averaged over the second half of this simulation. The obtained average value was used in the subsequent steps in an NVT ensemble as the cell vector. After 1 ns of equilibration, 50 ns of production run was performed.

The analysis of the trajectories was performed by the TRAVIS program.[17] The surface coverages were calculated by using the Voronoi tessellation-based domain analysis function of TRAVIS.[18] In this latter approach, the Voronoi cells are produced for each atom in the system. The area of the shared interfaces of these Voronoi cells are also summed for each kind of pairs: plastic-lipid, plastic-water, plastic-plastic interfaces. The total surface of the nanoparticle is obtained by the total surface of the Voronoi cells of the atoms that constitute the plastic minus the plastic-plastic shared interface. Accordingly, aggregation should result in the decrease of the total surface of the particle, while decomposition should be observable through its increase.
